# Frequentist and Bayesian Quantum Phase Estimation

**DOI:** 10.3390/e20090628

**Published:** 2018-08-23

**Authors:** Yan Li, Luca Pezzè, Manuel Gessner, Zhihong Ren, Weidong Li, Augusto Smerzi

**Affiliations:** 1 Institute of Theoretical Physics and Department of Physics, State Key Laboratory of Quantum Optics and Quantum Optics Devices, Collaborative Innovation Center of Extreme Optics, Shanxi University, Taiyuan 030006, China; 2 QSTAR, INO-CNR and LENS, Largo Enrico Fermi 2, 50125 Firenze, Italy

**Keywords:** quantum metrology, Bayesian estimation, parameter estimation

## Abstract

Frequentist and Bayesian phase estimation strategies lead to conceptually different results on the state of knowledge about the true value of an unknown parameter. We compare the two frameworks and their sensitivity bounds to the estimation of an interferometric phase shift limited by quantum noise, considering both the cases of a fixed and a fluctuating parameter. We point out that frequentist precision bounds, such as the Cramér–Rao bound, for instance, do not apply to Bayesian strategies and vice versa. In particular, we show that the Bayesian variance can overcome the frequentist Cramér–Rao bound, which appears to be a paradoxical result if the conceptual difference between the two approaches are overlooked. Similarly, bounds for fluctuating parameters make no statement about the estimation of a fixed parameter.

## 1. Introduction

The estimation of a phase shift using interferometric techniques is at the core of metrology and sensing [1,2,3]. Applications range from the definition of the standard of time [4] to the detection of gravitational waves [5,6]. The general problem can be concisely stated as the search for optimal strategies to minimize the phase estimation uncertainty. The noise that limits the achievable phase sensitivity can have a “classical” or a “quantum” nature. Classical noise originates from the coupling of the interferometer with some external source of disturbance, like seismic vibrations, parasitic magnetic fields or from incoherent interactions within the interferometer. Such noise can, in principle, be arbitrarily reduced, e.g., by shielding the interferometer from external noise or by tuning interaction parameters to ensure a fully coherent time evolution. The second source of uncertainty has an irreducible quantum origin [7,8]. Quantum noise cannot be fully suppressed, even in the idealized case of the creation and manipulation of pure quantum states. Using classically-correlated probe states, it is possible to reach the so-called shot noise or standard quantum limit, which is the limiting factor for the current generation of interferometers and sensors [9,10,11,12]. Strategies involving probe states characterized by squeezed quadratures [13] or entanglement between particles [14,15,16,17,18,19] are able to overcome the shot noise, the ultimate quantum bound being the so-called Heisenberg limit. Quantum noise reduction in phase estimation has been demonstrated in several proof-of-principle experiments with atoms and photons [20,21].

There is a vast amount of literature dealing with the parameter estimation problem that has been mostly developed following two different approaches [22,23,24]: frequentist and Bayesian. Both approaches have been investigated in the context of quantum phase estimation [18,20,25,26,27,28,29,30,31] and implemented/tested experimentally [32,33,34,35,36]. They build on conceptually different meanings attached to the word “probability” and their respective results provide conceptually different information on the estimated parameters and their uncertainties.

In the limit of a large number of repeated measurements, the sensitivity reached by the frequentist and Bayesian methods generally agree: this fact has very often induced the belief that the two paradigms can be interchangeably used in the phase estimation theory without acknowledging their irreconcilable nature. Overlooking these differences is not only conceptually inconsistent but can even create paradoxes, as, for instance, the existence of ultimate bounds in sensitivity proven in one paradigm that can be violated in the other.

In this manuscript, we directly compare the frequentist and the Bayesian parameter estimation theory. We study different sensitivity bounds obtained in the two frameworks and highlight the conceptual differences between the two. Besides the asymptotic regime of many repeated measurements, we also study bounds that are relevant for small samples. In particular, we show that the Bayesian variance can overcome the frequentist Cramér–Rao bound. The Cramér–Rao bound is a mathematical theorem providing the highest possible sensitivity in a phase estimation problem. The fact that the Bayesian sensitivity can be higher than the Cramér–Rao bound is therefore paradoxical. The paradox is solved by clarifying the conceptual differences between the frequentist and the Bayesian approaches, which therefore cannot be directly compared. Such difference should be considered when discussing theoretical and experimental figures of merit in interferometric phase estimation.

Our results are illustrated with a simple test model [37,38]. We consider *N* qubits with basis states |0〉 and |1〉, initially prepared in a (generalized) GHZ state $|\mathrm{GHZ}\rangle ={(|0\rangle }^{⊗N}+{|1\rangle }^{⊗N})/\sqrt{2}$, with all particles being either in |1〉 or in |0〉. The phase-encoding is a rotation of each qubit in the Bloch sphere $|0\rangle \to e^{-i\theta /2}|0\rangle$ and $|1\rangle \to e^{+i\theta /2}|1\rangle$, which transforms the |GHZ〉 state into $|\mathrm{GHZ}\left(\theta \right)\rangle ={(e^{-iN\theta /2}|0\rangle }^{⊗N}+e^{+iN\theta /2}{|1\rangle }^{⊗N})/\sqrt{2}$. The phase is estimated by measuring the parity ${\left(-1\right)}^{N_{0}}$, where $N_{0}$ is the number of particles in the state |0〉 [37,39,40,41]. The parity measurement has two possible results μ=±1 that are conditioned by the “true value of the phase shift” $\theta_{0}$ with probability $p\left(\pm 1|\theta_{0}\right)=\left(1\pm \cos\left(N\theta_{0}\right)\right)/2$. The probability to observe the sequence of results $\mu =\{\mu_{1},\mu_{2},\ldots ,\mu_{m}\}$ in *m* independent repetitions of the experiment (with same probe state and phase encoding transformation) is

(1) $$p\left(\mu |\theta_{0}\right)=\prod_{i=1}^{m}p\left(\mu_{i}|\theta_{0}\right)={\left(\frac{1+\cos\left(N\theta_{0}\right)}{2}\right)}^{m_{+}}{\left(\frac{1-\cos\left(N\theta_{0}\right)}{2}\right)}^{m_{-}},$$

where $m_{\pm }$ is the number of the observed results ±1, respectively. Notice that $p(\mu |\theta_{0})$ is the conditional probability for the measurement outcome μ, given that the true value of the phase shift is $\theta_{0}$ (which we consider to be unknown in the estimation protocol). Equation (1) provides the probability that will be used in the following sections for the case N=2 and $\theta_{0}\in \left[0,\pi /2\right]$. Section 2 and Section 3 deal with the case where $\theta_{0}$ has a fixed value and in Section 4 we discuss precision bounds for a fluctuating phase shift.

## 2. Frequentist Approach

In the frequentist paradigm, the phase (assumed having a fixed but unknown value $\theta_{0}$) is estimated via an arbitrarily chosen function of the measurement results, $\theta_{\mathrm{est}}\left(\mu \right)$, called the estimator. Typically, $\theta_{\mathrm{est}}\left(\mu \right)$ is chosen by maximizing the likelihood of the observed data (see below). The estimator, being a function of random outcomes, is itself a random variable. It is characterized by a statistical distribution that has an objective, measurable character. The relative frequency with which the event $\theta_{\mathrm{est}}$ occurs converges to a probability asymptotically with the number of repeated experimental trials.

### 2.1. Frequentist Risk Functions

Statistical fluctuations of the data reflect the *statistical uncertainty* of the estimation. This is quantified by the variance,

(2) $${\left(\Delta^{2}\theta_{\mathrm{est}}\right)}_{\mu |\theta_{0}}=\sum_{\mu }{\left(\theta_{\mathrm{est}}\left(\mu \right)-{\langle \theta_{\mathrm{est}}\rangle }_{\mu |\theta_{0}}\right)}^{2}p\left(\mu |\theta_{0}\right),$$

around the mean value ${\langle \theta_{\mathrm{est}}\rangle }_{\mu |\theta_{0}}=\sum_{\mu }\theta_{\mathrm{est}}\left(\mu \right)p\left(\mu |\theta_{0}\right)$, the sum extending over all possible measurement sequences (for fixed $\theta_{0}$ and *m*). An important class is that of *locally unbiased* estimators, namely those satisfying ${\langle \theta_{\mathrm{est}}\rangle }_{\mu |\theta_{0}}=\theta_{0}$ and $\frac{d{\langle \theta_{\mathrm{est}}\rangle }_{\mu |\theta }}{d\theta }{|}_{\theta =\theta_{0}}=1$ (see, for instance, [42]). An estimator is unbiased if and only if it is locally unbiased at every $\theta_{0}$.

The quality of the estimator can also be quantified by the mean square error (MSE) [23]

(3) $$\mathrm{MSE}{\left(\theta_{\mathrm{est}}\right)}_{\mu |\theta_{0}}=\sum_{\mu }{\left(\theta_{\mathrm{est}}\left(\mu \right)-\theta_{0}\right)}^{2}p\left(\mu |\theta_{0}\right),$$

giving the deviation of $\theta_{\mathrm{est}}$ from the true value of the phase shift $\theta_{0}$. It is related to Equation (2) by the relation

(4) $$\mathrm{MSE}{\left(\theta_{\mathrm{est}}\right)}_{\mu |\theta_{0}}={\left(\Delta^{2}\theta_{\mathrm{est}}\right)}_{\mu |\theta_{0}}+{\left({\langle \theta_{\mathrm{est}}\rangle }_{\mu |\theta_{0}}-\theta_{0}\right)}^{2}.$$

In the frequentist approach, often the variance is not considered as a proper way to quantify the goodness of an estimator. For instance, an estimator that always gives the same value independently of the measurement outcomes is strongly biased: it has zero variance but a large MSE that does not scale with the number of repeated measurements. Notice that the MSE cannot be accessed from the experimentally available data since the true value $\theta_{0}$ is unknown. In this sense, only the fluctuations of $\theta_{\mathrm{est}}$ around its mean value, i.e., the variance ${\left(\Delta^{2}\theta_{\mathrm{est}}\right)}_{\mu |\theta_{0}}$, have experimental relevance. For unbiased estimators, Equations (2) and (4) coincide. In general, since the bias term in Equation (4) is never negative, $\mathrm{MSE}{\left(\theta_{\mathrm{est}}\right)}_{\mu |\theta_{0}}\geq {\left(\Delta^{2}\theta_{\mathrm{est}}\right)}_{\mu |\theta_{0}}$ and any lower bound on ${\left(\Delta^{2}\theta_{\mathrm{est}}\right)}_{\mu |\theta_{0}}$ automatically provides a lower bound on $\mathrm{MSE}{\left(\theta_{\mathrm{est}}\right)}_{\mu |\theta_{0}}$ but not vice versa. In the following section, we therefore limit our attention to bounds on ${\left(\Delta^{2}\theta_{\mathrm{est}}\right)}_{\mu |\theta_{0}}$. The distinction between the two quantities becomes more important in the case of a fluctuating phase shift $\theta_{0}$, where the bias can affect the corresponding bounds in different ways. We will see this explicitly in Section 4.

### 2.2. Frequentist Bounds on Phase Sensitivity

#### 2.2.1. Barankin Bound

The Barankin bound (BB) provides the tightest lower bound to the variance (2) [43]. It can be proven to be always (for any *m*) saturable, in principle, by a specific local (i.e., dependent of $\theta_{0}$) estimator and measurement observable. Of course, since the estimator that saturates the BB depends on the true value of the parameter (which is unknown), the bound is of not much use in practice. Nevertheless, the BB plays a central role, from the theoretical point of view, as it provides a hierarchy of weaker bounds which can be used in practice with estimators that are asymptotically unbiased. The BB can be written as [44]

(5) $${\left(\Delta^{2}\theta_{\mathrm{est}}\right)}_{\mu |\theta_{0}}\geq \Delta^{2}\theta_{\mathrm{BB}}\equiv \underset{\theta_{i},a_{i},n}{\sup}\frac{{\left\{\sum_{i=1}^{n}a_{i}\left[{\langle \theta_{\mathrm{est}}\rangle }_{\mu |\theta_{i}}-{\langle \theta_{\mathrm{est}}\rangle }_{\mu |\theta_{0}}\right]\right\}}^{2}}{\sum_{\mu }{\left[\sum_{i=1}^{n}a_{i}L\left(\mu |\theta_{i},\theta_{0}\right)\right]}^{2}p\left(\mu |\theta_{0}\right)},$$

where $L\left(\mu |\theta_{i},\theta \right)=p\left(\mu |\theta_{i}\right)/p\left(\mu |\theta \right)$ is generally indicated as likelihood ratio and the supremum is taken over *n* parameters $a_{i}\in R$, which are arbitrary real numbers, and $\theta_{i}$, which are arbitrary phase values in the parameter domain. For unbiased estimators, we can replace ${\langle \theta_{\mathrm{est}}\rangle }_{\mu |\theta_{i}}=\theta_{i}$ for all *i* and the BB becomes independent of the estimator:

(6) $${\left(\Delta^{2}\theta_{\mathrm{est}}\right)}_{\mu |\theta_{0}}\geq \Delta^{2}\theta_{\mathrm{BB}}^{\mathrm{ub}}\equiv \underset{\theta_{i},a_{i},n}{\sup}\frac{{\left\{\sum_{i=1}^{n}a_{i}\left[\theta_{i}-\theta_{0}\right]\right\}}^{2}}{\sum_{\mu }{\left[\sum_{i=1}^{n}a_{i}L\left(\mu |\theta_{i},\theta_{0}\right)\right]}^{2}p\left(\mu |\theta_{0}\right)}.$$

A derivation of the BB is presented in Appendix A.

The explicit calculation of $\Delta^{2}\theta_{\mathrm{BB}}$ is impractical in most applications due to the number of free variables that must be optimized. However, the BB provides a strict hierarchy of bounds of increasing complexity that can be of great practical importance. Restricting the number of variables in the optimization can provide local lower bounds that are much simpler to determine at the expense of not being saturable in general, namely, for an arbitrary number of measurements. Below, we demonstrate the following hierarchy of bounds:

(7) $${\left(\Delta^{2}\theta_{\mathrm{est}}\right)}_{\mu |\theta_{0}}\geq \Delta^{2}\theta_{\mathrm{BB}}\geq \Delta^{2}\theta_{\mathrm{EChRB}}\geq \Delta^{2}\theta_{\mathrm{ChRB}}\geq \Delta^{2}\theta_{\mathrm{CRLB}},$$

where $\Delta^{2}\theta_{\mathrm{CRLB}}$ is the Cramér–Rao lower bound (CRLB) [45,46] and $\Delta^{2}\theta_{\mathrm{ChRB}}$ is the Hammersley–Chapman–Robbins bound (ChRB) [47,48]. We will also introduce a novel extended version of the ChRB, indicated as $\Delta^{2}\theta_{\mathrm{EChRB}}$.

#### 2.2.2. Cramér–Rao Lower Bound and Maximum Likelihood Estimator

The CRLB is the most common frequentist bound in parameter estimation. It is given by [45,46]:

(8) $$\Delta^{2}\theta_{\mathrm{CRLB}}=\frac{{\left(\frac{d{\langle \theta_{\mathrm{est}}\rangle }_{\mu |\theta_{0}}}{d\theta_{0}}\right)}^{2}}{mF(\theta_{0})}.$$

The inequality ${\left(\Delta^{2}\theta_{\mathrm{est}}\right)}_{\mu |\theta_{0}}\geq \Delta^{2}\theta_{\mathrm{CRLB}}$ is obtained by differentiating ${\langle \theta_{\mathrm{est}}\rangle }_{\mu |\theta_{0}}$ with respect to $\theta_{0}$ and using a Cauchy–Schwarz inequality:

(9) $${\left(\frac{d{\langle \theta_{\mathrm{est}}\rangle }_{\mu |\theta_{0}}}{d\theta_{0}}\right)}^{2}={\left(\sum_{\mu }(\theta_{\mathrm{est}}\left(\mu \right)-{\langle \theta_{\mathrm{est}}\rangle }_{\mu |\theta_{0}})\frac{dp(\mu |\theta_{0})}{d\theta_{0}}\right)}^{2}\leq mF\left(\theta_{0}\right){\left(\Delta^{2}\theta_{\mathrm{est}}\right)}_{\mu |\theta_{0}},$$

where we have used $\sum_{\mu }\frac{dp(\mu |\theta_{0})}{d\theta_{0}}=0$ and $\sum_{\mu }\frac{1}{p(\mu |\theta_{0})}{\left(\frac{\partial p(\mu |\theta )}{\partial \theta }{|}_{\theta_{0}}\right)}^{2}=m\sum_{\mu }\frac{1}{p(\mu |\theta_{0})}{\left(\frac{\partial p(\mu |\theta )}{\partial \theta }{|}_{\theta_{0}}\right)}^{2}$ valid for *m* independent measurements, and

(10) $$F\left(\theta_{0}\right)=\sum_{\mu }\frac{1}{p(\mu |\theta_{0})}{\left(\frac{\partial p(\mu |\theta )}{\partial \theta }{|}_{\theta_{0}}\right)}^{2}$$

is the Fisher information. The equality ${\left(\Delta^{2}\theta_{\mathrm{est}}\right)}_{\mu |\theta_{0}}=\Delta^{2}\theta_{\mathrm{CRLB}}$ is achieved if and only if

(11) $$\theta_{\mathrm{est}}\left(\mu \right)-{\langle \theta_{\mathrm{est}}\rangle }_{\mu |\theta_{0}}=\lambda_{\theta_{0}}\frac{d\log p(\mu |\theta_{0})}{d\theta_{0}},$$

with $\lambda_{\theta_{0}}$ a parameter independent of μ (while it may depend on $\theta_{0}$). Noticing that $\frac{d{\langle \theta_{\mathrm{est}}\rangle }_{\mu |\theta_{0}}}{d\theta_{0}}=\sum_{\mu }\left(\theta_{\mathrm{est}}\left(\mu \right)-f\left(\theta_{0}\right)\right)\frac{dp(\mu |\theta_{0})}{d\theta_{0}}$, the CRLB can be straightforwardly generalized to any function $f(\theta_{0})$ independent of μ. In particular, choosing $f\left(\theta_{0}\right)=\theta_{0}$, we can directly prove that $\mathrm{MSE}{\left(\theta_{\mathrm{est}}\right)}_{\mu |\theta_{0}}\geq \Delta^{2}\theta_{\mathrm{CRLB}}$, which also depends on the bias.

Asymptotically in *m*, the saturation of Equation (8) is obtained for the maximum likelihood estimator (MLE) [22,23,49]. This is the value $\theta_{\mathrm{MLE}}\left(\mu \right)$ that maximizes the likelihood $p(\mu |\theta_{0})$ (as a function of the parameter $\theta_{0}$) for the observed measurement sequence μ,

(12) $$\theta_{\mathrm{MLE}}\left(\mu \right)\equiv \arg\max_{\theta_{0}}\left\{p\left(\mu |\theta_{0}\right)\right\}.$$

For a sufficiently large sample size *m* (in the central limit), independently of the probability distribution $p(\mu |\theta_{0})$, the MLE becomes normally distributed [18,22,23,49]:

(13) $$p\left(\theta_{\mathrm{MLE}}|\theta_{0}\right)=\sqrt{\frac{mF\left(\theta_{0}\right)}{2\pi }}e^{-\frac{mF\left(\theta_{0}\right)}{2}{\left(\theta_{0}-\theta_{\mathrm{MLE}}\right)}^{2}}\;\left(m\gg 1\right),$$

with mean given by the true value $\theta_{0}$ and variance equal to the inverse of the Fisher information. The MLE is well defined provided that there is a unique maximum in the considered phase interval. In the case of Equation (1), this condition is fulfilled provided that one restrict the phase domain to [0,π/(2N)] for instance.

**Figure 1:**
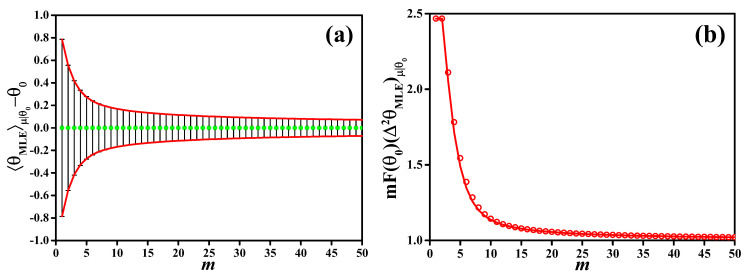
(**a**) Bias ${\langle \theta_{\mathrm{MLE}}\rangle }_{\mu |\theta_{0}}-\theta_{0}$ (green dots) as function of *m* with error bars ${\left(\Delta \theta_{\mathrm{MLE}}\right)}_{\mu |\theta_{0}}$. The red lines are $\pm \Delta \theta_{\mathrm{CRLB}}=\pm \left|d{\langle \theta_{\mathrm{MLE}}\rangle }_{\mu |\theta_{0}}/d\theta_{0}\right|/\sqrt{mF(\theta_{0})}$; (**b**) variance of the maximum likelihood estimator multiplied by the Fisher information, $mF\left(\theta_{0}\right){\left(\Delta^{2}\theta_{\mathrm{MLE}}\right)}_{\mu |\theta_{0}}$ (red circles), as a function of the sample size *m*. It is compared to the bias ${\left(d{\langle \theta_{\mathrm{MLE}}\rangle }_{\mu |\theta_{0}}/d\theta_{0}\right)}^{2}$ (red dashed line). We recall that $\theta_{0}=\pi /4$ and $F(\theta_{0})=4$ here.

In Figure 1, we plot the results of a maximum likelihood analysis for the example considered in this manuscript. In this case, the MLE is readily calculated and given by $\theta_{\mathrm{MLE}}\left(\mu \right)=\frac{1}{2}\arccos\left(\frac{m_{+}-m_{-}}{m_{+}+m_{-}}\right)$, and the Fisher information is $F\left(\theta_{0}\right)=N^{2}$, independent of $\theta_{0}$ (we recall that N=2 in our example). In Figure 1a we plot the bias ${\langle \theta_{\mathrm{MLE}}\rangle }_{\mu |\theta_{0}}-\theta_{0}$ (dots) as a function of *m*, for $\theta_{0}=\pi /4$. Error bars are $\pm \Delta \theta_{\mathrm{CRLB}}$. Notice that ${\langle \theta_{\mathrm{MLE}}\rangle }_{\mu |\theta_{0}}=\theta_{0}$ for every *m*. This does not mean that the estimator is locally unbiased: indeed, the derivative $d{\langle \theta_{\mathrm{MLE}}\rangle }_{\mu |\theta_{0}}/d\theta_{0}$ [see panel (b)] is different from 1 for every value of *m*. We have $d{\langle \theta_{\mathrm{MLE}}\rangle }_{\mu |\theta_{0}}/d\theta_{0}\to 1$ asymptotically in *m*. In Figure 1b, we plot $mF\left(\theta_{0}\right){\left(\Delta^{2}\theta_{\mathrm{MLE}}\right)}_{\mu |\theta_{0}}$ as a function of the number of independent measurements *m* (red dots). This quantity is compared to $mF\left(\theta_{0}\right)\Delta^{2}\theta_{\mathrm{CRLB}}={\left(d{\langle \theta_{\mathrm{MLE}}\rangle }_{\mu |\theta_{0}}/d\theta_{0}\right)}^{2}$ (red line). With increasing sample size *m*, ${\left(\Delta^{2}\theta_{\mathrm{MLE}}\right)}_{\mu |\theta_{0}}\to 1/\left(mF(\theta_{0})\right)$ corresponding to the CRLB for unbiased estimators.

#### 2.2.3. Hammersley–Chapman–Robbins Bound

The ChRB is obtained from Equation (5) by taking n=2, $a_{1}=1,a_{2}=-1$, $\theta_{1}=\theta_{0}+\lambda$, $\theta_{2}=\theta_{0}$, and can be written as [47,48]

(14) $$\Delta^{2}\theta_{\mathrm{ChRB}}=\underset{\lambda }{\sup}\frac{{\left({\langle \theta_{\mathrm{est}}\rangle }_{\mu |\theta_{0}+\lambda }-{\langle \theta_{\mathrm{est}}\rangle }_{\mu |\theta_{0}}\right)}^{2}}{\sum_{\mu }\frac{p{\left(\mu |\theta_{0}+\lambda \right)}^{2}}{p(\mu |\theta_{0})}-1}.$$

Clearly, restricting the number of parameters in the optimization in Equation (5) leads to a less strict bound. We thus have $\Delta^{2}\theta_{\mathrm{BB}}\geq \Delta^{2}\theta_{\mathrm{ChRB}}$. For unbiased estimators, we obtain

(15) $$\Delta^{2}\theta_{\mathrm{ChRB}}^{\mathrm{ub}}=\underset{\lambda }{\sup}\frac{\lambda^{2}}{\sum_{\mu }\frac{p{\left(\mu |\theta_{0}+\lambda \right)}^{2}}{p(\mu |\theta_{0})}-1}.$$

**Figure 2:**
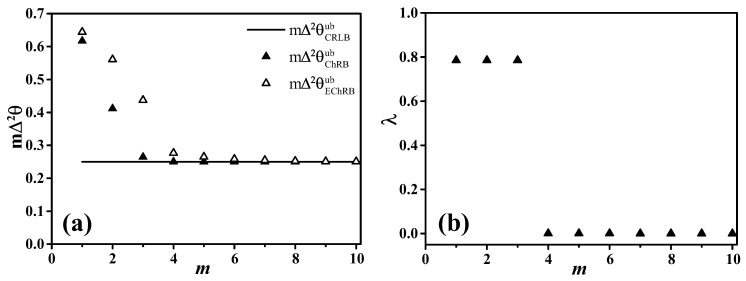
(**a**) comparison between unbiased frequentist bounds for the example considered in this manuscript, Equation (1): the CRLB $m\Delta^{2}\theta_{\mathrm{CRLB}}^{\mathrm{ub}}=1/F\left(\theta_{0}\right)$ (black line), the Hammersley–Chapman–Robbins bound $m\Delta^{2}\theta_{\mathrm{ChRB}}^{\mathrm{ub}}$ (Equation (15), filled triangles) and the extended Hammersley–Chapman–Robbins bound $m\Delta^{2}\theta_{\mathrm{EChRB}}^{\mathrm{ub}}$ (Equation (18), empty triangles); (**b**) values of λ achieving the supremum in Equation (15), as a function of *m*.

Furthermore, the supremum over λ on the right side of Equation (14) is always larger or equal to its limit λ→0:

(16) $$\begin{array}{cc} \underset{\lambda }{\sup}\frac{{\left({\langle \theta_{\mathrm{est}}\rangle }_{\mu |\theta_{0}+\lambda }-{\langle \theta_{\mathrm{est}}\rangle }_{\mu |\theta_{0}}\right)}^{2}}{\sum_{\mu }\frac{p{\left(\mu |\theta_{0}+\lambda \right)}^{2}}{p(\mu |\theta_{0})}-1} & \geq \lim_{\lambda \to 0}\frac{{\left({\langle \theta_{\mathrm{est}}\rangle }_{\mu |\theta_{0}+\lambda }-{\langle \theta_{\mathrm{est}}\rangle }_{\mu |\theta_{0}}\right)}^{2}}{\sum_{\mu }\frac{p{\left(\mu |\theta_{0}+\lambda \right)}^{2}}{p(\mu |\theta_{0})}-1}=\frac{{\left(\frac{d{\langle \theta_{\mathrm{est}}\rangle }_{\mu |\theta_{0}}}{d\theta_{0}}\right)}^{2}}{m\sum_{\mu }\frac{1}{p(\mu |\theta_{0})}{\left(\frac{dp(\mu |\theta_{0})}{d\theta_{0}}\right)}^{2}}, \end{array}$$

provided that the derivatives on the right-hand side exist. We thus recover the CRLB as a limiting case of the ChRB. The ChRB is always stricter than the CRLB and we obtain the last inequality in the chain (7). Notice that the CRLB requires the probability distribution $p(\mu |\theta_{0})$ to be differentiable [24]—a condition that can be dropped for the ChRB and the more general BB. Even if the distribution is regular, the above derivation shows that the ChRB, and more generally the BB, provide tighter error bounds than the CRLB. With increasing *n*, the BB becomes tighter and tighter and the CRLB represents the weakest bound in this hierarchy, which can be observed in Figure 2a. Next, we determine a stricter bound in this hierarchy.

#### 2.2.4. Extended Hammersley–Chapman–Robbins Bound

We obtain the extended Hammersley–Chapman–Robbins bound (EChRB) as a special case of Equation (5), by taking n=3, $a_{1}=1$, $a_{2}=A$, $a_{3}=-1$, $\theta_{1}=\theta_{0}+\lambda_{1}$, $\theta_{2}=\theta_{0}+\lambda_{2}$, and $\theta_{3}=\theta_{0}$, giving

(17) $$\Delta^{2}\theta_{\mathrm{EChRB}}=\underset{\lambda_{1},\lambda_{2},A}{\sup}\frac{{\left({\langle \theta_{\mathrm{est}}\rangle }_{\mu |\theta_{0}+\lambda_{1}}+A{\langle \theta_{\mathrm{est}}\rangle }_{\mu |\theta_{0}+\lambda_{2}}-\left(1+A\right){\langle \theta_{\mathrm{est}}\rangle }_{\mu |\theta_{0}}\right)}^{2}}{\sum_{\mu }\frac{{\left[p\left(\mu |\theta_{0}+\lambda_{1}\right)-p\left(\mu |\theta_{0}\right)+Ap\left(\mu |\theta_{0}+\lambda_{2}\right)\right]}^{2}}{p(\mu |\theta_{0})}},$$

where the supremum is taken over all possible $\lambda_{1},\lambda_{2}\in N$ and A∈R. Since the ChRB is obtained from Equation (17) in the specific case A=0, we have that $\Delta^{2}\theta_{\mathrm{EChRB}}\geq \Delta^{2}\theta_{\mathrm{ChRB}}$. For unbiased estimators, we obtain

(18) $$\Delta^{2}\theta_{\mathrm{EChRB}}^{\mathrm{ub}}=\underset{\lambda_{1},\lambda_{2},A}{\sup}\frac{{\left(\lambda_{1}+A\lambda_{2}\right)}^{2}}{\sum_{\mu }\frac{{\left[p\left(\mu |\theta_{0}+\lambda_{1}\right)-p\left(\mu |\theta_{0}\right)+Ap\left(\mu |\theta_{0}+\lambda_{2}\right)\right]}^{2}}{p(\mu |\theta_{0})}}.$$

In Figure 2a, we compare the different bounds for unbiased estimators and for the example considered in the manuscript: the CRLB (black line), the ChRB (filled triangles) and the EChRB (empty triangles), satisfying the chain of inequalities (7). In Figure 2b, we show the values of λ in Equation (15) for which the supremum is achieved in our case.

## 3. Bayesian Approach

The Bayesian approach makes use of the Bayes–Laplace theorem, which can be very simply stated and proved. The joint probability of two stochastic variables μ and θ is symmetric: p(μ,θ)=p(μ|θ)p(θ)=p(θ|μ)p(μ)=p(θ,μ), where p(θ) and p(μ) are the marginal distributions, obtained by integrating the joint probability over one of the two variables, while p(μ|θ) and p(θ|μ) are conditional distributions.

We recall that in a phase inference problem, the set of measurement results μ is generated by a fixed and unknown value $\theta_{0}$ according to the likelihood $p(\mu |\theta_{0})$. In the Bayesian approach to the estimation of $\theta_{0}$, one introduces a random variable θ and uses the Bayes–Laplace theorem to define the conditional probability

(19) $$p_{\mathrm{post}}\left(\theta |\mu \right)=\frac{p\left(\mu |\theta \right)p_{\mathrm{pri}}\left(\theta \right)}{p_{\mathrm{mar}}\left(\mu \right)}.$$

The posterior probability $p_{\mathrm{post}}\left(\theta |\mu \right)$ provides a degree of belief, or plausibility, that $\theta_{0}=\theta$ (i.e., that θ is the true value of the phase), in the light of the measurement data μ [50]. In Equation (19), the prior distribution $p_{\mathrm{pri}}\left(\theta \right)$ expresses the a priori state of knowledge on θ, p(μ|θ) is the likelihood that is determined by the quantum mechanical measurement postulate, e.g., as in Equation (1), and the marginal probability $p_{\mathrm{mar}}\left(\mu \right)=\int_{a}^{b}d\theta \;p\left(\theta ,\mu \right)$ is obtained through the normalization for the posterior, where *a* and *b* are boundaries of the phase domain. The posterior probability $p_{\mathrm{post}}\left(\theta |\mu \right)$ describes the current knowledge about the random variable θ based on the available information, i.e., the measurement results μ.

### 3.1. Noninformative Prior

In the Bayesian approach, the information on θ provided by the posterior probability always depends on the prior distribution $p_{\mathrm{pri}}\left(\theta \right)$. It is possible to account for the available a priori information on θ by choosing a prior distribution accordingly. However, if no a priori information is available, it is not obvious how to choose a “noninformative” prior [51]. The flat prior $p_{\mathrm{pri}}\left(\theta \right)=\mathrm{const}$ was first introduced by Laplace to express the absence of information on θ[51]. However, this prior would not be flat for other functions of θ and, in the complete absence of a priori information, it seems unreasonable that some information is available for different parametrizations of the problem. To see this, recall that a transformation of variables requires that $p_{\mathrm{pri}}\left(\varphi \right)=p_{\mathrm{pri}}\left(\theta \right)\left|df^{-1}\left(\varphi \right)/d\varphi \right|$ for any function φ=f(θ). Hence, if $p_{\mathrm{pri}}\left(\theta \right)$ is flat, one obtains that $p_{\mathrm{pri}}\left(\varphi \right)=\left|df^{-1}\left(\varphi \right)/d\varphi \right|$ is, in general, not flat.

Notice that $p_{\mathrm{pri}}\left(\theta \right)\propto \sqrt{F(\theta )}$—called Jeffreys prior [52,53]—where F(θ) is the Fisher information (10), remains invariant under re-parametrization. For arbitrary transformations φ=f(θ), the Fisher information obeys the transformation property $F\left(\varphi \right)=F\left(\theta \right){\left(d\theta /d\varphi \right)}^{2}=F\left(\theta \right){\left(df^{-1}\left(\varphi \right)/d\varphi \right)}^{2}$. Therefore, if $p_{\mathrm{pri}}\left(\theta \right)\propto \sqrt{F(\theta )}$ and we perform the change of variable φ=f(θ), then the transformation property of the Fisher information ensures that $p_{\mathrm{pri}}\left(\varphi \right)=p_{\mathrm{pri}}\left(\theta \right)\left|df^{-1}\left(\varphi \right)/d\varphi \right|\propto \sqrt{F(\varphi )}$. Notice that, as in our case, the Fisher information F(θ) may actually be independent of θ. In this case, the invariance property does not imply that Jeffreys prior is flat for arbitrary re-parametrizations φ=f(θ), instead, $\sqrt{F(\varphi )}=\left|df^{-1}\left(\varphi \right)/d\varphi \right|$.

### 3.2. Posterior Bounds

From the posterior probability (19), we can provide an estimate $\theta_{\mathrm{BL}}\left(\mu \right)$ of $\theta_{0}$. This can be the maximum a posteriori, $\theta_{\mathrm{BL}}\left(\mu \right)=\arg\max_{\theta }\;p_{\mathrm{post}}\left(\theta |\mu \right)$, which coincides with the maximum likelihood Equation (12) when the prior is flat, $p_{\mathrm{pri}}\left(\theta \right)=\mathrm{const}$, or the mean of the distribution, $\theta_{\mathrm{BL}}\left(\mu \right)=\int_{a}^{b}d\theta \;\theta \;p_{\mathrm{post}}\left(\theta |\mu \right)$.

With the Bayesian approach, it is possible to provide a confidence interval around the estimator, given an arbitrary measurement sequence μ, even with a single measurement. The variance

(20) $${\left(\Delta^{2}\theta_{\mathrm{BL}}\left(\mu \right)\right)}_{\theta |\mu }=\int_{a}^{b}d\theta \;p_{\mathrm{post}}\left(\theta |\mu \right){\left(\theta -\theta_{\mathrm{BL}}\left(\mu \right)\right)}^{2}$$

can be taken as a measure of fluctuation of our degree of belief around $\theta_{\mathrm{BL}}\left(\mu \right)$. There is no such concept in the frequentist paradigm. The Bayesian posterior variance ${\left(\Delta^{2}\theta_{\mathrm{BL}}\left(\mu \right)\right)}_{\theta |\mu }$ and the frequentist variance ${\left(\Delta^{2}\theta_{\mathrm{BL}}\right)}_{\mu |\theta_{0}}$ have entirely different operational meanings. Equation (20) provides a degree of plausibility that $\theta_{\mathrm{BL}}\left(\mu \right)=\theta_{0}$, given the measurement results μ. There is no notion of bias in this case. On the other hand, the quantity ${\left(\Delta^{2}\theta_{\mathrm{BL}}\right)}_{\mu |\theta_{0}}$ measures the statistical fluctuations of $\theta_{\mathrm{BL}}\left(\mu \right)$ when repeating the sequence of *m* measurements infinitely many times.

#### Ghosh Bound

In the following, we derive a lower bound to Equation (20) first introduced by Ghosh [54]. Using $\int_{a}^{b}d\theta \;p_{\mathrm{post}}\left(\theta |\mu \right)=1,$ we have

(21) $$\begin{array}{cc} \int_{a}^{b}d\theta \;\left(\theta -\theta_{\mathrm{BL}}\left(\mu \right)\right)\frac{dp_{\mathrm{post}}\left(\theta |\mu \right)}{d\theta } & ={\left.p_{\mathrm{post}}\left(\theta |\mu \right)\left(\theta -\theta_{\mathrm{BL}}\left(\mu \right)\right)\right|}_{a}^{b}-\int_{a}^{b}d\theta \;p_{\mathrm{post}}\left(\theta |\mu \right)=f\left(\mu ,a,b\right)-1,\; \end{array}$$

where $f\left(\mu ,a,b\right)=bp_{\mathrm{post}}\left(b|\mu \right)-ap_{\mathrm{post}}\left(a|\mu \right)-\theta_{\mathrm{BL}}\left(\mu \right)\left(p_{\mathrm{post}}\left(b|\mu \right)-p_{\mathrm{post}}\left(a|\mu \right)\right)$ depends on the value of the posterior distribution calculated at the boundaries. If $p_{\mathrm{pri}}\left(a\right)=p_{\mathrm{pri}}\left(b\right)=0$, we have $f\left(\mu ,a,b\right)=0$. Analogously with the derivation of the (frequenstist) CRLB, we exploit the Cauchy–Schwarz inequality,

$$\left(\int_{a}^{b}d\theta {\left(\frac{dp_{\mathrm{post}}\left(\theta |\mu \right)}{d\theta }\right)}^{2}\frac{1}{p_{\mathrm{post}}\left(\theta |\mu \right)}\right)\left(\int_{a}^{b}d\theta \;p_{\mathrm{post}}\left(\theta |\mu \right){\left(\theta -\theta_{\mathrm{BL}}\left(\mu \right)\right)}^{2}\right)\geq {\left(f\left(\mu ,a,b\right)-1\right)}^{2},$$

leading to ${\left(\Delta^{2}\theta_{\mathrm{BL}}\left(\mu \right)\right)}_{\theta |\mu }\geq \Delta^{2}\theta_{\mathrm{GB}}\left(\mu \right)$, where [54]

(22) $$\Delta^{2}\theta_{\mathrm{GB}}\left(\mu \right)=\frac{{\left(f\left(\mu ,a,b\right)-1\right)}^{2}}{\int_{a}^{b}d\theta \;\frac{1}{p_{\mathrm{post}}\left(\theta |\mu \right)}{\left(\frac{dp_{\mathrm{post}}\left(\theta |\mu \right)}{d\theta }\right)}^{2}}.$$

The above bound is a function of the specific measurement sequence μ and depends on $\int_{a}^{b}d\theta \;\frac{1}{p_{\mathrm{post}}\left(\theta |\mu \right)}{\left(\frac{dp_{\mathrm{post}}\left(\theta |\mu \right)}{d\theta }\right)}^{2}$ that we can identify as a “Fisher information of the posterior distribution”. The Ghosh bound is saturated if and only if

(23) $$\theta -\theta_{\mathrm{BL}}\left(\mu \right)=\lambda_{\mu }\frac{d\log p(\theta |\mu )}{d\theta },$$

where $\lambda_{\mu }$ does not depend on θ while it may depend on μ.

### 3.3. Average Posterior Bounds

While Equation (20) depends on the specific μ, it is natural to consider its average over all possible measurement sequences at fixed $\theta_{0}$ and *m*, weighted by the likelihood $p(\mu |\theta_{0})$:

(24) $${\left(\Delta^{2}\theta_{\mathrm{BL}}\right)}_{\mu ,\theta |\theta_{0}}=\sum_{\mu }{\left(\Delta^{2}\theta_{\mathrm{BL}}\left(\mu \right)\right)}_{\theta |\mu }\;p\left(\mu |\theta_{0}\right)=\sum_{\mu }\int_{a}^{b}d\theta \;p\left(\theta ,\mu |\theta_{0}\right){\left(\theta -\theta_{\mathrm{BL}}\left(\mu \right)\right)}^{2},$$

which we indicate as average Bayesian posterior variance, where $p\left(\theta ,\mu |\theta_{0}\right)=p_{\mathrm{post}}\left(\theta |\mu \right)p\left(\mu |\theta_{0}\right)$.

We would be tempted to compare the average posterior sensitivity ${\left(\Delta^{2}\theta_{\mathrm{BL}}\right)}_{\mu ,\theta |\theta_{0}}$ to the frequentist Cramér–Rao bound $\Delta^{2}\theta_{\mathrm{CRLB}}$. However, because of the different operational meanings of the frequentist and the Bayesian paradigms, there is no reason for Equation (24) to fulfill the Cramér–Rao bound: indeed, it does not, as we show below.

#### Likelihood-Averaged Ghosh Bound

A lower bound to Equation (24) is obtained by averaging the Ghosh bound Equation (22) over the likelihood function. We have ${\left(\Delta^{2}\theta_{\mathrm{BL}}\right)}_{\mu ,\theta |\theta_{0}}\geq \Delta^{2}\theta_{\mathrm{aGB}}$, where [18]

(25) $$\Delta^{2}\theta_{\mathrm{aGB}}=\sum_{\mu }\frac{{\left(f\left(\mu ,a,b\right)-1\right)}^{2}}{\int_{a}^{b}d\theta \;\frac{1}{p_{\mathrm{post}}\left(\theta |\mu \right)}{\left(\frac{\partial p_{\mathrm{post}}\left(\theta |\mu \right)}{\partial \theta }\right)}^{2}}\;p\left(\mu |\theta_{0}\right).$$

This likelihood-averaged Ghosh bound is independent of μ because of the statistical average.

### 3.4. Numerical Comparison of Bayesian and Frequentist Phase Estimation

**Figure 3:**
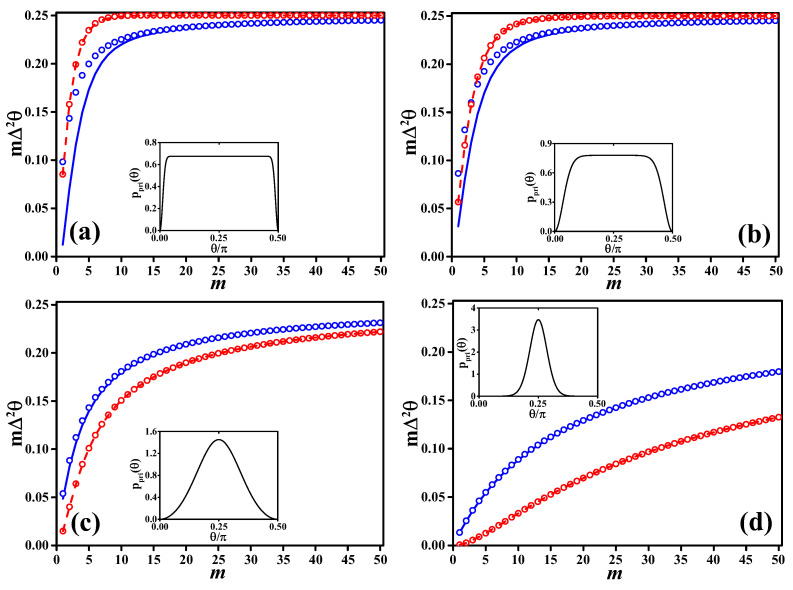
Comparisons of phase estimation variance as a function of the sample size for Bayesian and frequentist data analysis under different prior distributions, (**a**) α=−100, (**b**) α=−10, (**c**) α=1, (**d**) α=10. In all figures, Red circles (frequentist) are $m{\left(\Delta^{2}\theta_{\mathrm{BL}}\right)}_{\mu |\theta_{0}}$, the red dashed line is the Cramér-Rao lower bound $m\Delta^{2}\theta_{\mathrm{CRLB}}$, Equation (8). Blue circles (Bayesian) are $m{\left(\Delta^{2}\theta_{\mathrm{BL}}\right)}_{\mu ,\theta |\theta_{0}}$, the blue solid line is the likelihood-averaged Ghosh bound $m\Delta^{2}\theta_{\mathrm{aGB}}$, Equation (25). The inset in each panel is $p_{\mathrm{pri}}\left(\theta \right)$, Equation (26), for the corresponding values of α.

In the numerical calculations shown in Figure 3, we consider a Bayesian estimator given by $\theta_{\mathrm{BL}}\left(\mu \right)=\int_{a}^{b}d\theta \;\theta \;p_{\mathrm{post}}\left(\theta |\mu \right)$ with prior distributions

(26) $$p_{\mathrm{pri}}\left(\theta \right)=\frac{2}{\pi }\frac{e^{\alpha \sin{\left(2\theta \right)}^{2}}-1}{e^{\alpha /2}I_{0}\left(\alpha /2\right)-1},$$

where $I_{0}\left(\alpha \right)$ is the modified Bessel function of the first kind. This choice of prior distribution can continuously turn from a peaked function to a flat one when changing α, while being differentiable in the full phase interval. The more negative is α, the more $p_{\mathrm{pri}}\left(\theta \right)$ broadens in [0,π/2]. In particular, in the limit α→−∞, the prior approaches the flat distribution, which in our case coincides with Jeffreys prior since the Fisher information is independent of θ. In the limit α=0, the prior is given by $\lim_{\alpha \to 0}p_{\mathrm{pri}}\left(\theta \right)=4\sin{\left(2\theta \right)}^{2}/\pi$. For positive values of α, the larger α, the more peaked is $p_{\mathrm{pri}}\left(\theta \right)$ around $\theta_{0}=\pi /4$. In particular $p_{\mathrm{pri}}\left(\theta \right)\approx e^{-4\alpha {\left(\theta -\pi /4\right)}^{2}}/\sqrt{\pi /4\alpha }$ for α≫1. Equation (26) is normalized to one for $\theta \in [0,\frac{\pi }{2}]$. In the inset of the different panels of Figure 3, we plot $p_{\mathrm{pri}}\left(\theta \right)$ for α=−100 [panel (a)], α=−10 (b), α=1 (c) and α=10 (d).

In Figure 3, we plot, as a function of *m*, the posterior variance ${\left(\Delta^{2}\theta_{\mathrm{BL}}\right)}_{\mu ,\theta |\theta_{0}}$ (blue circles) that, as expected, is always larger than the likelihood-averaged Ghosh bound Equation (25) (solid blue lines). For comparison, we also plot the frequentist variance ${\left(\Delta^{2}\theta_{\mathrm{BL}}\right)}_{\mu |\theta_{0}}=\sum_{\mu }{\left(\theta_{\mathrm{BL}}\left(\mu \right)-{\langle \theta_{\mathrm{BL}}\rangle }_{\mu |\theta_{0}}\right)}^{2}p\left(\mu |\theta_{0}\right)$ (red dots) around the mean value ${\langle \theta_{\mathrm{BL}}\rangle }_{\mu |\theta_{0}}=\sum_{\mu }\theta_{\mathrm{BL}}\left(\mu \right)p\left(\mu |\theta_{0}\right)$ of the estimator. This quantity obeys the Cramér–Rao theorem ${\left(\Delta^{2}\theta_{\mathrm{BL}}\right)}_{\mu |\theta_{0}}\geq \Delta^{2}\theta_{\mathrm{CRLB}}$ and the more general chain of inequalities (7). This is confirmed in the figure where we show $\Delta^{2}\theta_{\mathrm{CRLB}}={\left|d{\langle \theta_{\mathrm{BL}}\rangle }_{\mu |\theta_{0}}/d\theta_{0}\right|}^{2}/\left(mF(\theta_{0})\right)$ (red line). Notice that, when the prior narrows around $\theta_{0}$, the variance ${\left(\Delta^{2}\theta_{\mathrm{BL}}\right)}_{\mu |\theta_{0}}$ decreases, but, at the same time, the estimator becomes more and more biased, i.e., $|d{\langle \theta_{\mathrm{BL}}\rangle }_{\mu |\theta_{0}}/d\theta_{0}|$ decreases as well (note indeed that the red dashed line is proportional to $|d{\langle \theta_{\mathrm{BL}}\rangle }_{\mu |\theta_{0}}/d\theta_{0}|{}^{2}$).

Interestingly, in Figure 3, we clearly see that the Bayesian posterior variance ${\left(\Delta^{2}\theta_{\mathrm{BL}}\right)}_{\mu ,\theta |\theta_{0}}$ and the likelihood-averaged Ghosh bound may stay in some cases below the (frequentist) $\Delta^{2}\theta_{\mathrm{CRLB}}$ [see panels (a) and (b)], even if the prior is almost flat. The discrepancy with the CRLB is remarkable and can be quite large for small values of *m*. Still, there is no contradiction since ${\left(\Delta^{2}\theta_{\mathrm{BL}}\right)}_{\mu ,\theta |\theta_{0}}$ and ${\left(\Delta^{2}\theta_{\mathrm{BL}}\right)}_{\mu |\theta_{0}}$ have different operational meanings and interpretations. They both respect their corresponding sensitivity bounds.

Asymptotically in the number of measurements *m*, the Ghosh bound as well as its likelihood average converge to the Cramér–Rao bound. Indeed, it is well known that in this limit the posterior probability becomes a Gaussian centered at the true value of the phase shift and with variance given by the inverse of the Fisher information,

(27) $$p_{\mathrm{post}}\left(\theta |\mu \right)=\sqrt{\frac{mF(\theta_{0})}{2\pi }}e^{-\frac{mF(\theta_{0})}{2}{\left(\theta -\theta_{0}\right)}^{2}},\;\left(m\gg 1\right),$$

a result known as Laplace–Bernstein–von Mises theorem [18,23,55]. By replacing Equation (27) into Equation (22), we recover a posterior variance given by $1/\left(mF(\theta_{0})\right)$.

## 4. Bounds for Random Parameters

In this section, we derive bounds of phase sensitivity obtained when $\theta_{0}$ is a random variable distributed according to $p(\theta_{0})$. Operationally, this corresponds to the situation where $\theta_{0}$ remains fixed (but unknown) when collecting a single sequence of *m* measurements μ. In between measurement sequences, $\theta_{0}$ fluctuates according to $p(\theta_{0})$.

### 4.1. Frequentist Risk Functions for Random Parameters

Let us first consider the frequentist estimation of a fluctuating parameter $\theta_{0}$ with the estimator $\theta_{\mathrm{est}}$. The mean sensitivity obtained by averaging ${\left(\Delta^{2}\theta_{\mathrm{est}}\right)}_{\mu |\theta_{0}}$, Equation (3), over $p(\theta_{0})$ is

(28) $$\begin{array}{ccc} {\left(\Delta^{2}\theta_{\mathrm{est}}\right)}_{\mu ,\theta_{0}} & = & \int_{a}^{b}d\theta_{0}{\left(\Delta^{2}\theta_{\mathrm{est}}\right)}_{\mu |\theta_{0}}p\left(\theta_{0}\right)\\ & = & \sum_{\mu }\int_{a}^{b}d\theta_{0}\;p\left(\mu |\theta_{0}\right)p\left(\theta_{0}\right){\left({\langle \theta_{\mathrm{est}}\rangle }_{\mu |\theta_{0}}-\theta_{\mathrm{est}}\left(\mu \right)\right)}^{2}\\ & = & \sum_{\mu }\int_{a}^{b}d\theta_{0}\;p\left(\mu ,\theta_{0}\right){\left({\langle \theta_{\mathrm{est}}\rangle }_{\mu |\theta_{0}}-\theta_{\mathrm{est}}\left(\mu \right)\right)}^{2}, \end{array}$$

where μ and $\theta_{0}$ are both random variables and we have used $p\left(\mu |\theta_{0}\right)p\left(\theta_{0}\right)=p\left(\mu ,\theta_{0}\right)$.

An averaged risk function for the efficiency of the estimator is given by averaging the mean square error (3) over $p(\theta_{0})$, leading to

(29) $$\mathrm{MSE}{\left(\theta_{\mathrm{est}}\right)}_{\mu ,\theta_{0}}=\int d\theta_{0}\mathrm{MSE}{\left(\theta_{\mathrm{est}}\right)}_{\mu |\theta_{0}}p\left(\theta_{0}\right)=\int d\theta_{0}\sum_{\mu }{\left(\theta_{\mathrm{est}}\left(\mu \right)-\theta_{0}\right)}^{2}p\left(\mu ,\theta_{0}\right).$$

Analogously to Equation (4), we can write

(30) $$\mathrm{MSE}{\left(\theta_{\mathrm{est}}\right)}_{\mu ,\theta_{0}}={\left(\Delta^{2}\theta_{\mathrm{est}}\right)}_{\mu ,\theta_{0}}+\int d\theta_{0}{\left({\langle \theta_{\mathrm{est}}\rangle }_{\mu |\theta_{0}}-\theta_{0}\right)}^{2}p\left(\theta_{0}\right).$$

In the following, we derive lower bounds for both ${\left(\Delta^{2}\theta_{\mathrm{est}}\right)}_{\mu ,\theta_{0}}$ and $\mathrm{MSE}{\left(\theta_{\mathrm{est}}\right)}_{\mu ,\theta_{0}}$. Notice that bounds on ${\left(\Delta^{2}\theta_{\mathrm{est}}\right)}_{\mu ,\theta_{0}}$ hold also for $\mathrm{MSE}{\left(\theta_{\mathrm{est}}\right)}_{\mu ,\theta_{0}}$ due to $\mathrm{MSE}{\left(\theta_{\mathrm{est}}\right)}_{\mu ,\theta_{0}}\geq {\left(\Delta^{2}\theta_{\mathrm{est}}\right)}_{\mu ,\theta_{0}}$. Nevertheless, bounds on the average the mean square error are widely used (and are often called Bayesian bounds [56]) since they can be expressed independently of the bias.

### 4.2. Bounds on the Mean Square Error

We first consider bounds on $\mathrm{MSE}{\left(\theta_{\mathrm{est}}\right)}_{\mu ,\theta_{0}}$, Equation (29), for arbitrary estimators.

#### 4.2.1. Van Trees Bound

It is possible to derive a general lower bound on the mean square error (29) based on the following assumptions:

1. $\frac{\partial p(\mu ,\theta_{0})}{\partial \theta_{0}}$ and $\frac{\partial^{2}p\left(\mu ,\theta_{0}\right)}{\partial \theta_{0}^{2}}$ are absolutely integrable with respect to μ and $\theta_{0}$;
2. $p\left(a\right)\xi \left(a\right)-p\left(b\right)\xi \left(b\right)=0$, where $\xi \left(\theta_{0}\right)=\sum_{\mu }\left(\theta_{\mathrm{est}}\left(\mu \right)-\theta_{0}\right)p\left(\mu |\theta_{0}\right)$.

Multiplying $\xi (\theta_{0})$ by $p(\theta_{0})$ and differentiating with respect to $\theta_{0}$, we have

$$\frac{\partial p\left(\theta_{0}\right)\xi \left(\theta_{0}\right)}{\partial \theta_{0}}=\sum_{\mu }\left(\theta_{\mathrm{est}}\left(\mu \right)-\theta_{0}\right)\frac{\partial p(\mu ,\theta_{0})}{\partial \theta_{0}}-p\left(\theta_{0}\right).$$

Integrating over $\theta_{0}$ in the range of [a,b] and considering the above properties, we find

(31) $$\sum_{\mu }\int_{a}^{b}d\theta_{0}\left(\theta_{\mathrm{BL}}\left(\mu \right)-\theta_{0}\right)\frac{\partial p(\mu ,\theta_{0})}{\partial \theta_{0}}=1.$$

Finally, using the Cauchy–Schwarz inequality, we arrive at $\mathrm{MSE}{\left(\theta_{\mathrm{est}}\right)}_{\mu ,\theta_{0}}\geq \Delta^{2}\theta_{\mathrm{VTB}}$, where

(32) $$\Delta^{2}\theta_{\mathrm{VTB}}=\frac{1}{\sum_{\mu }\int_{a}^{b}d\theta_{0}\frac{1}{p(\mu ,\theta_{0})}{\left(\frac{\partial p(\mu ,\theta_{0})}{\partial \theta_{0}}\right)}^{2}}$$

is generally indicated as Van Trees bound [24,56,57]. The equality holds if and only if

(33) $$\theta_{\mathrm{est}}\left(\mu \right)-\theta_{0}=\lambda \frac{d\log p(\mu ,\theta_{0})}{d\theta_{0}},$$

where λ does not depend on $\theta_{0}$ and μ. It is easy to show that

(34) $$\sum_{\mu }\int_{a}^{b}d\theta_{0}\frac{1}{p(\mu ,\theta_{0})}{\left(\frac{\partial p(\mu ,\theta_{0})}{\partial \theta_{0}}\right)}^{2}=m\int_{a}^{b}d\theta_{0}\;p\left(\theta_{0}\right)F\left(\theta_{0}\right)+\int_{a}^{b}d\theta_{0}\frac{1}{p(\theta_{0})}{\left(\frac{\partial p(\theta_{0})}{\partial \theta_{0}}\right)}^{2},$$

where the first term is the Fisher information $F(\theta_{0})$, defined by Equation (10), averaged over $p(\theta_{0})$, and the second term can be interpreted as a Fisher information of the prior [24]. Asymptotically in the number of measurements *m* and for regular distributions $p(\theta_{0})$, the first term in Equation (34) dominates over the second one.

#### 4.2.2. Ziv–Zakai Bound

A further bound on $\mathrm{MSE}{\left(\theta_{\mathrm{est}}\right)}_{\mu ,\theta_{0}}$ can be derived by mapping the phase estimation problem to a continuous series of binary hypothesis testing problems. A detailed derivation of the Ziv–Zakai bound [24,58,59] is provided in Appendix B. The final result reads $\mathrm{MSE}{\left(\theta_{\mathrm{est}}\right)}_{\mu ,\theta_{0}}\geq \Delta^{2}\theta_{\mathrm{ZZB}}$, where

(35) $$\Delta^{2}\theta_{\mathrm{ZZB}}=\frac{1}{2}\int dh\;h\int d\theta_{0}\left(p\left(\theta_{0}\right)+p\left(\theta_{0}+h\right)\right)P_{\min}\left(\theta_{0},\theta_{0}+h\right),$$

and

(36) $$P_{\min}\left(\theta_{0},\theta_{0}+h\right)=\frac{1}{2}\left(1-\sum_{\mu }\left|\frac{p\left(\theta_{0}\right)p\left(\mu |\theta_{0}\right)}{p\left(\theta_{0}\right)+p\left(\theta_{0}+h\right)}-\frac{p\left(\theta_{0}+h\right)p\left(\mu |\theta_{0}+h\right)}{p\left(\theta_{0}\right)+p\left(\theta_{0}+h\right)}\right|\right)$$

is the minimum error probability of the binary hypothesis testing problem. This bound has been adopted for quantum phase estimation in Ref. [26]. To this end, the probability $P_{\min}\left(\theta_{0},\theta_{0}+h\right)$ can be maximized over all possible quantum measurements, which leads to the trace distance [7]. As the optimal measurement may depend on $\theta_{0}$ and *h*, the bound (35), which involves integration over all values of $\theta_{0}$ and *h*, is usually not saturable. We remark that the trace distance also defines a saturable frequentist bound for a different risk function than the variance [60].

### 4.3. Bounds on the Average Estimator Variance

We now consider bounds on ${\left(\Delta^{2}\theta_{\mathrm{est}}\right)}_{\mu ,\theta_{0}}$, Equation (28), for arbitrary estimators.

#### 4.3.1. Average CRLB

Taking the average over $p(\theta_{0})$ of Equation (7), we obtain a chain of bounds for ${\left(\Delta^{2}\theta_{\mathrm{est}}\right)}_{\mu ,\theta_{0}}$. In particular, in its simplest form, we have ${\left(\Delta^{2}\theta_{\mathrm{est}}\right)}_{\mu ,\theta_{0}}\geq \Delta^{2}\theta_{\mathrm{aCRLB}}$, where

(37) $$\Delta^{2}\theta_{\mathrm{aCRLB}}=\int_{a}^{b}d\theta_{0}\frac{{\left(\frac{d{\langle \theta_{\mathrm{est}}\rangle }_{\mu |\theta_{0}}}{d\theta_{0}}\right)}^{2}}{mF(\theta_{0})}p\left(\theta_{0}\right)$$

is the average CRLB.

#### 4.3.2. Van Trees Bound for the Average Estimator Variance

We can derive a general lower bound for the variance (28) by following the derivation of the Van Trees bound, which was discussed in Section 4.2.1. In contrast to the standard Van Trees bound for the mean square error, here the bias enters explicitly. Defining $\xi \left(\theta_{0}\right)=\sum_{\mu }\left(\theta_{\mathrm{est}}\left(\mu \right)-{\langle \theta_{\mathrm{est}}\rangle }_{\mu |\theta_{0}}\right)p\left(\mu |\theta_{0}\right)$ and assuming the same requirements as in the derivation of the Van Trees bound for the MSE, we arrive at

$$\sum_{\mu }\int_{a}^{b}d\theta_{0}(\theta_{\mathrm{est}}\left(\mu \right)-{\langle \theta_{\mathrm{est}}\rangle }_{\mu |\theta_{0}})\frac{\partial p(\mu ,\theta_{0})}{\partial \theta_{0}}=\int_{a}^{b}d\theta_{0}\frac{d{\langle \theta_{\mathrm{est}}\rangle }_{\mu |\theta_{0}}}{d\theta_{0}}p\left(\theta_{0}\right).$$

Finally, a Cauchy–Schwarz inequality gives ${\left(\Delta^{2}\theta_{\mathrm{est}}\right)}_{\mu ,\theta_{0}}\geq \Delta^{2}\theta_{\mathrm{fVTB}}$, where

(38) $$\Delta^{2}\theta_{\mathrm{fVTB}}=\frac{{\left(\int_{a}^{b}d\theta_{0}\frac{d{\langle \theta_{\mathrm{est}}\rangle }_{\mu |\theta_{0}}}{d\theta_{0}}p\left(\theta_{0}\right)\right)}^{2}}{\sum_{\mu }\int_{a}^{b}d\theta_{0}\frac{1}{p(\mu ,\theta_{0})}{\left(\frac{\partial p(\mu ,\theta_{0})}{\partial \theta_{0}}\right)}^{2}},$$

with equality if and only if

(39) $$\theta_{\mathrm{est}}\left(\mu \right)-{\langle \theta_{\mathrm{est}}\rangle }_{\mu |\theta_{0}}=\lambda \frac{d\log p(\mu ,\theta_{0})}{d\theta_{0}},$$

where λ is independent of $\theta_{0}$ and μ.

We can compare Equation (38) with the average CRLB Equation (37). We find

$$\int_{a}^{b}d\theta_{0}\frac{{\left(\frac{d{\langle \theta_{\mathrm{est}}\rangle }_{\mu |\theta_{0}}}{d\theta_{0}}\right)}^{2}}{mF(\theta_{0})}p\left(\theta_{0}\right)\geq \frac{{\left(\int_{a}^{b}d\theta_{0}\frac{d{\langle \theta_{\mathrm{est}}\rangle }_{\mu |\theta_{0}}}{d\theta_{0}}p\left(\theta_{0}\right)\right)}^{2}}{m\int_{a}^{b}d\theta_{0}p\left(\theta_{0}\right)F\left(\theta_{0}\right)}\geq \frac{{\left(\int_{a}^{b}d\theta_{0}|\frac{d{\langle \theta_{\mathrm{est}}\rangle }_{\mu |\theta_{0}}}{d\theta_{0}}|p\left(\theta_{0}\right)\right)}^{2}}{\sum_{\mu }\int_{a}^{b}d\theta_{0}\frac{1}{p(\mu ,\theta_{0})}{\left(\frac{\partial p(\mu ,\theta_{0})}{\partial \theta_{0}}\right)}^{2}},$$

where in the first step we use Jensen’s inequality, and the second step follows from Equation (34) which implies $m\int_{a}^{b}d\theta_{0}p\left(\theta_{0}\right)F\left(\theta_{0}\right)\leq \sum_{\mu }\int_{a}^{b}d\theta_{0}\frac{1}{p(\mu ,\theta_{0})}{\left(\frac{\partial p(\mu ,\theta_{0})}{\partial \theta_{0}}\right)}^{2}$ since $\int_{a}^{b}d\theta_{0}\frac{1}{p(\theta_{0})}{\left(\frac{dp(\theta_{0})}{d\theta_{0}}\right)}^{2}\geq 0$.

We thus arrive at

(40) $${\left(\Delta^{2}\theta_{\mathrm{est}}\right)}_{\mu ,\theta_{0}}\geq \Delta^{2}\theta_{\mathrm{aCRLB}}\geq \Delta^{2}\theta_{\mathrm{fVTB}},$$

which is valid for generic estimators.

### 4.4. Bayesian Framework for Random Parameters

The Bayesian posterior variance, ${\left(\Delta^{2}\theta_{\mathrm{BL}}\right)}_{\mu ,\theta |\theta_{0}}$, Equation (24), averaged over $p(\theta_{0})$ is

(41) $$\begin{array}{ccc} {\left(\Delta^{2}\theta_{\mathrm{BL}}\right)}_{\mu ,\theta ,\theta_{0}} & = & \int_{a}^{b}d\theta_{0}{\left(\Delta^{2}\theta_{\mathrm{BL}}\right)}_{\mu ,\theta |\theta_{0}}\;p\left(\theta_{0}\right)\\ & = & \sum_{\mu }\int_{a}^{b}d\theta \int_{a}^{b}d\theta_{0}\;p_{\mathrm{post}}\left(\theta |\mu \right)p\left(\mu |\theta_{0}\right)p\left(\theta_{0}\right){\left(\theta -\theta_{\mathrm{BL}}\left(\mu \right)\right)}^{2}\\ & = & \sum_{\mu }\int_{a}^{b}d\theta \;p_{\mathrm{post}}\left(\theta |\mu \right)p\left(\mu \right){\left(\theta -\theta_{\mathrm{BL}}\left(\mu \right)\right)}^{2}, \end{array}$$

where $p\left(\mu \right)=\int_{a}^{b}d\theta_{0}\;p\left(\mu |\theta_{0}\right)p\left(\theta_{0}\right)$ is the average probability to observe μ taking into account fluctuations of $\theta_{0}$.

A bound on Equation (41) can be obtained by averaging Equation (25) over $p(\theta_{0})$, or, equivalently, averaging the Ghosh bound, Equation (22), over p(μ). We obtain the average Ghosh bound for random parameters $\theta_{0}$, ${\left(\Delta^{2}\theta_{\mathrm{BL}}\right)}_{\mu ,\theta ,\theta_{0}}\geq \Delta^{2}\theta_{\mathrm{aGBr}}$, where

(42) $$\begin{array}{ccc} \Delta^{2}\theta_{\mathrm{aGBr}} & = & \int_{a}^{b}d\theta_{0}\sum_{\mu }\frac{{\left(f\left(\mu ,a,b\right)-1\right)}^{2}}{\int_{a}^{b}d\theta \frac{1}{p_{\mathrm{post}}\left(\theta |\mu \right)}{\left(\frac{dp_{\mathrm{post}}\left(\theta |\mu \right)}{d\theta }\right)}^{2}}p\left(\mu |\theta_{0}\right)p\left(\theta_{0}\right)\\ & = & \sum_{\mu }\frac{{\left(f\left(\mu ,a,b\right)-1\right)}^{2}}{\int_{a}^{b}d\theta \;\frac{1}{p_{\mathrm{post}}\left(\theta |\mu \right)}{\left(\frac{dp_{\mathrm{post}}\left(\theta |\mu \right)}{d\theta }\right)}^{2}}p\left(\mu \right). \end{array}$$

The bound holds for any prior $p_{\mathrm{pri}}\left(\theta \right)$ and is saturated if and only if, for every value of μ, there exists a $\lambda_{\mu }$ such that Equation (23) holds.

#### Bayesian Bounds

In Equation (41), the prior used to define the posterior $p_{\mathrm{post}}\left(\theta |\mu \right)$ via the Bayes–Laplace theorem is arbitrary. In general, such a prior $p_{\mathrm{pri}}\left(\theta \right)$ is different from the statistical distribution of $\theta_{0}$, which can be unknown. If $p(\theta_{0})$ is known, then one can use it as a prior in the Bayesian posterior probability, i.e., $p_{\mathrm{pri}}\left(\theta \right)=p\left(\theta_{0}\right)$. In this specific case, we have $p_{\mathrm{mar}}\left(\mu \right)=p\left(\mu \right)$, and thus $p_{\mathrm{post}}\left(\theta |\mu \right)p\left(\mu \right)=p_{\mathrm{post}}\left(\theta |\mu \right)p_{\mathrm{mar}}\left(\mu \right)=p\left(\mu ,\theta \right)$. In other words, for this specific choice of prior, the physical joint probability $p(\mu ,\theta_{0})$ of random variables $\theta_{0}$ and μ coincides with the Bayesian p(μ,θ). Equation (41) thus simplifies to

(43) $${\left(\Delta^{2}\theta_{\mathrm{BL}}\right)}_{\mu ,\theta }=\sum_{\mu }\int_{a}^{b}d\theta \;p\left(\mu ,\theta \right){\left(\theta -\theta_{\mathrm{BL}}\left(\mu \right)\right)}^{2}.$$

Notice that this expression is mathematically equivalent to the frequentist average mean square error (29) if we replace θ with $\theta_{0}$ and $\theta_{\mathrm{BL}}\left(\mu \right)$ with $\theta_{\mathrm{est}}\left(\mu \right)$. This means that precision bounds for Equation (29), e.g., the Van Trees and Ziv–Zakai bounds can also be applied to Equation (43). These bounds are indeed often referred to as “Bayesian bounds” (see Ref. [24]).

**Figure 4:**
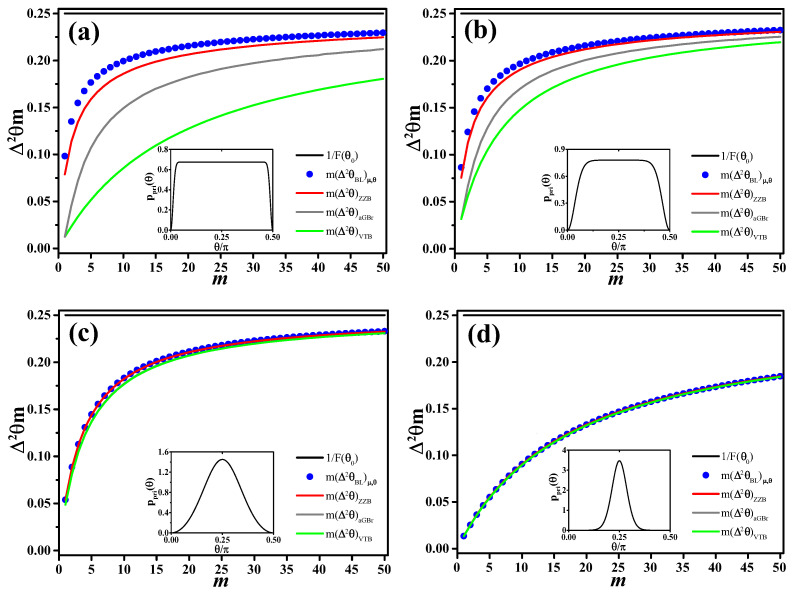
Comparisons of average posterior Bayesian variance, $m{\left(\Delta^{2}\theta_{\mathrm{BL}}\right)}_{\mu ,\theta }$ (dots), as a function of the sample size *m* under different prior distributions, (**a**) α=−100, (**b**) α=−10, (**c**) α=1, (**d**) α=10. This variance is compared to to the average Ghosh bound for random parameters $m(\Delta^{2}\theta_{\mathrm{aGBr}})$ (grey line), the Van Trees bound $m(\Delta^{2}\theta_{\mathrm{VTB}})$ (green line), the Ziv–Zakai bound $m(\Delta^{2}\theta_{\mathrm{ZZB}})$ (red line) and $1/F(\theta_{0})$ (black horizontal line). The inset in each panel is the prior $p_{\mathrm{pri}}\left(\theta \right)$, Equation (26), for the corresponding values of α.

We emphasize that the average over the marginal distribution $p_{\mathrm{mar}}\left(\mu \right)$, which connects Equations (24) and (43), has operational meaning if we consider that $\theta_{0}$ is a random variable distributed according to $p(\theta_{0})$, and p(θ) is used as prior in the Bayes–Laplace theorem to define a posterior distribution. In this case, and under the condition f(μ,a,b)=0 (for instance if the prior distribution vanishes at the borders of the phase domain), using Jensen’s inequality, we find

(44) $$\begin{array}{ccc} \Delta^{2}\theta_{\mathrm{aGBr}} & = & \sum_{\mu }\frac{p(\mu )}{\int_{a}^{b}d\theta \;\frac{1}{p_{\mathrm{post}}\left(\theta |\mu \right)}{\left(\frac{dp_{\mathrm{post}}\left(\theta |\mu \right)}{d\theta }\right)}^{2}}\\ & \geq & \frac{1}{\sum_{\mu }p\left(\mu \right)\;\int_{a}^{b}d\theta \;\frac{1}{p_{\mathrm{post}}\left(\theta |\mu \right)}{\left(\frac{dp_{\mathrm{post}}\left(\theta |\mu \right)}{d\theta }\right)}^{2}}\\ & = & \frac{1}{\sum_{\mu }\int_{a}^{b}d\theta \;\frac{1}{p(\theta ,\mu )}{\left(\frac{\partial p(\theta ,\mu )}{\partial \theta }\right)}^{2}}, \end{array}$$

which coincides with the Van Trees bound discussed above. We thus find that the averaged Ghosh bound for random parameters (42) is sharper than the Van Trees bound (38):

(45) $${\left(\Delta^{2}\theta_{\mathrm{BL}}\right)}_{\mu ,\theta }\geq \Delta^{2}\theta_{\mathrm{aGBr}}\geq \Delta^{2}\theta_{\mathrm{VTB}},$$

which is also confirmed by the numerical data shown in Figure 4.

In Figure 4, we compare ${\left(\Delta^{2}\theta_{\mathrm{BL}}\right)}_{\mu ,\theta }$ with the various bounds discussed in this section. As $p(\theta_{0}),$ we consider the same prior (26) used in Figure 3. We observe that all bounds approach the Van Trees bound with increasing sharpness of the prior distribution. Asymptotically in the number of measurements *m*, all bounds converge to the Cramér–Rao bound.

## 5. Discussion and Conclusions

In this manuscript, we have clarified the differences between frequentist and Bayesian approaches to phase estimation. The two paradigms provide statistical results that have a different conceptual meaning and cannot be compared. We have also reviewed and discussed phase sensitivity bounds in the frequentist and Bayesian frameworks, when the true value of the phase shift $\theta_{0}$ is fixed or fluctuates. These bounds are summarized in Table 1.

In the frequentist approach, for a fixed $\theta_{0}$, the phase sensitivity is determined from the width of the probability distribution of the estimator. The physical content of the distribution is that, when repeating the estimation protocol, the obtained $\theta_{\mathrm{est}}\left(\mu \right)$ will fall, with a certain confidence, in an interval around the mean value ${\langle \theta_{\mathrm{est}}\rangle }_{\mu |\theta_{0}}$ (e.g., 68% of the times within a $2{\left(\Delta \theta_{\mathrm{est}}\right)}_{\mu |\theta_{0}}$ interval for a Gaussian distribution) that, for unbiased estimators, coincides with the true value of the phase shift.

In the Bayesian case, the posterior $p_{\mathrm{post}}\left(\theta |\mu \right)$ provides a degree of plausibility that the phase shift θ equals the interferometer phase $\theta_{0}$ when the data μ was obtained. This allows the Bayesian approach to provide statistical information for any number of measurements, even a single one. To be sure, this is not a sign of failure or superiority of one approach with respect to the other one, since the two frameworks manipulate conceptually different quantities. The experimentalist can choose to use one or both approaches, keeping in mind the necessity to clearly state the nature of the statistical significance of the reported results.

The two predictions converge asymptotically in the limit of a large number of measurements. This does not mean that in this limit the significance of the two approaches is interchangeable (it cannot be stated that in the limit of large repetition of the measurements, frequentist ad Bayesian provide the same results). In this respect, it is quite instructive to notice that the Bayesian 2σ confidence may be below that of the Cramér–Rao bound, as shown in Figure 3. This, at first sight, seems paradoxical, since the CRLB is a theorem about the minimum error achievable in parameter estimation theory. However, the CRLB is a frequentist bound and, again, the paradox is solved taking it into account that the frequentist and the Bayesian approaches provide information about different quantities.

Finally, a different class of estimation problems with different precision bounds is encountered if $\theta_{0}$ is itself a random variable. In this case, the frequentist bounds for the mean-square error (Van Trees, Ziv–Zakai) become independent of the bias, while those on the estimator variance are still functions of the bias. The Van Trees and Ziv–Zakai bounds can be applied to the Bayesian paradigm if the average of the posterior variance over the marginal distribution is the relevant risk function. This is only meaningful if the prior $p_{\mathrm{pri}}\left(\theta \right)$ that enters the Bayes–Laplace theorem coincides with the actual distribution $p(\theta_{0})$ of the phase shift $\theta_{0}$.

We conclude with a remark regarding the so-called Heisenberg limit, which is a saturable lower bound on the CRLB over arbitrary quantum states with a fixed number of particles. For instance, for a collection of *N* two-level systems, the CRLB can be further bounded by $\Delta \theta_{\mathrm{est}}\geq 1/\sqrt{mF(\theta_{0})}\geq 1/\left(\sqrt{m}N\right)$ [18,20]. This bound is often called the ultimate precision bound since no quantum state is able to achieve a tighter scaling than *N*. From the discussions presented in this article, it becomes apparent that Bayesian approaches (as discussed in Section 3) or precision bounds for random parameters (Section 4) are expected to lead to entirely different types of ‘ultimate’ lower bounds. Such bounds are interesting within the respective paradigm for which they are derived, but they cannot replace or improve the Heisenberg limit since they address fundamentally different scenarios that cannot be compared in general.

## Figures and Tables

**Table 1:** Frequentist vs Bayesian bounds for fixed and random parameters.

	Paradigm	Risk Function	Bounds		Remarks
$\theta_{0}$ fixed	Frequentist	${\left(\Delta^{2}\theta_{\mathrm{est}}\right)}_{\mu |\theta_{0}}$	BB	Equation (5)	hierarchy of bounds, Equation (7)
			EChRB	Equation (17)	
		$\mathrm{MSE}{\left(\theta_{\mathrm{est}}\right)}_{\mu |\theta_{0}}$	ChRB	Equation (14)	
			CRLB	Equation (8)	
	Bayesian	${\left(\Delta^{2}\theta_{\mathrm{BL}}\right)}_{\mu |\theta_{0}}$	GB	Equation (22)	function of μ
		${\left(\Delta^{2}\theta_{\mathrm{BL}}\right)}_{\mu ,\theta |\theta_{0}}$	aGB	Equation (25)	average over likelihood $p(\mu |\theta_{0})$
$\theta_{0}$ random	Frequentist	${\left(\Delta^{2}\theta_{\mathrm{est}}\right)}_{\mu ,\theta_{0}}$	aCRLB	Equation (37)	hierarchy of bounds, Equation (40)
			fVTB	Equation (38)	
		$\mathrm{MSE}{\left(\theta_{\mathrm{est}}\right)}_{\mu ,\theta_{0}}$	VTB	Equation (32)	bounds are independent of the bias
			ZZB	Equation (35)	
	Bayesian	${\left(\Delta^{2}\theta_{\mathrm{BL}}\right)}_{\mu ,\theta ,\theta_{0}}$	aGBr	Equation (42)	prior $p_{\mathrm{pri}}\left(\theta \right)$ and fluctuations $p(\theta_{0})$ arbitrary
		${\left(\Delta^{2}\theta_{\mathrm{BL}}\right)}_{\mu ,\theta }$	VTB	Equation (32)	prior $p_{\mathrm{pri}}\left(\theta \right)$ and fluctuations $p(\theta_{0})$ coincide
			ZZB	Equation (35)	hierarchy of bounds, Equation (45)

## Appendix A. Derivation of the Barankin Bound

Let $\theta_{\mathrm{est}}$ be an arbitrary estimator for θ. Its mean value

(A1) $${\langle \theta_{\mathrm{est}}\rangle }_{\mu |\theta }=\sum_{\mu }\theta_{\mathrm{est}}\left(\mu \right)p\left(\mu |\theta \right)$$

coincides with θ if and only if the estimator is unbiased (for arbitrary values of θ). In the following, we make no assumption about the bias of $\theta_{\mathrm{est}}$ and therefore do not replace ${\langle \theta_{\mathrm{est}}\rangle }_{\mu |\theta }$ by θ.

Introducing the likelihood ratio

(A2) $$L\left(\mu |\theta_{i},\theta_{0}\right)=\frac{p(\mu |\theta_{i})}{p(\mu |\theta_{0})}$$

under the condition $p(\mu |\theta_{0})>0$ for all μ, we obtain with Equation (A1) that

(A3) $$\sum_{\mu }\theta_{\mathrm{est}}\left(\mu \right)L\left(\mu |\theta_{i},\theta_{0}\right)p\left(\mu |\theta_{0}\right)={\langle \theta_{\mathrm{est}}\rangle }_{\mu |\theta_{i}},$$

for an arbitrary family of phase values $\theta_{1},\ldots ,\theta_{n}$ picked from the parameter domain. Furthermore, we have

(A4) $$\sum_{\mu }L\left(\mu |\theta_{i},\theta_{0}\right)p\left(\mu |\theta_{0}\right)=\sum_{\mu }p\left(\mu |\theta_{i}\right)=1$$

for all $\theta_{i}$. Multiplying both sides of Equation (A4) with ${\langle \theta_{\mathrm{est}}\rangle }_{\mu |\theta_{0}}$ and subtracting it from (A3) yields

(A5) $$\sum_{\mu }\left(\theta_{\mathrm{est}}\left(\mu \right)-{\langle \theta_{\mathrm{est}}\rangle }_{\mu |\theta_{0}}\right)L\left(\mu |\theta_{i},\theta_{0}\right)p\left(\mu |\theta_{0}\right)={\langle \theta_{\mathrm{est}}\rangle }_{\mu |\theta_{i}}-{\langle \theta_{\mathrm{est}}\rangle }_{\mu |\theta_{0}}.$$

Let us now pick a family of *n* finite coefficients $a_{1},\ldots ,a_{n}$. From Equation (A5), we obtain

(A6) $$\sum_{\mu }\left(\theta_{\mathrm{est}}\left(\mu \right)-{\langle \theta_{\mathrm{est}}\rangle }_{\mu |\theta_{0}}\right)\left(\sum_{i=1}^{n}a_{i}L\left(\mu |\theta_{i},\theta_{0}\right)\right)p\left(\mu |\theta_{0}\right)=\sum_{i=1}^{n}a_{i}\left({\langle \theta_{\mathrm{est}}\rangle }_{\mu |\theta_{i}}-{\langle \theta_{\mathrm{est}}\rangle }_{\mu |\theta_{0}}\right).$$

The Cauchy–Schwarz inequality now yields

(A7) $${\left(\sum_{i=1}^{n}a_{i}\left({\langle \theta_{\mathrm{est}}\rangle }_{\mu |\theta_{i}}-{\langle \theta_{\mathrm{est}}\rangle }_{\mu |\theta_{0}}\right)\right)}^{2}\leq {\left(\Delta^{2}\theta_{\mathrm{est}}\right)}_{\mu |\theta_{0}}\left(\sum_{\mu }{\left(\sum_{i=1}^{n}a_{i}L\left(\mu |\theta_{i},\theta_{0}\right)\right)}^{2}p\left(\mu |\theta_{0}\right)\right),$$

where

(A8) $${\left(\Delta^{2}\theta_{\mathrm{est}}\right)}_{\mu |\theta_{0}}=\sum_{\mu }{\left(\theta_{\mathrm{est}}\left(\mu \right)-{\langle \theta_{\mathrm{est}}\rangle }_{\mu |\theta_{0}}\right)}^{2}p\left(\mu |\theta_{0}\right)$$

is the variance of the estimator $\theta_{\mathrm{est}}$. We thus obtain

(A9) $${\left(\Delta^{2}\theta_{\mathrm{est}}\right)}_{\mu |\theta_{0}}\geq \frac{{\left(\sum_{i=1}^{n}a_{i}\left({\langle \theta_{\mathrm{est}}\rangle }_{\mu |\theta_{i}}-{\langle \theta_{\mathrm{est}}\rangle }_{\mu |\theta_{0}}\right)\right)}^{2}}{\sum_{\mu }{\left(\sum_{i=1}^{n}a_{i}L\left(\mu |\theta_{i},\theta_{0}\right)\right)}^{2}p\left(\mu |\theta_{0}\right)},$$

for all *n*, $a_{i}$, and $\theta_{i}$. The Barankin bound then follows by taking the supremum over these variables.

## Appendix B. Derivation of the Ziv–Zakai Bound

Derivations of the Ziv–Zakai bound can be found in the literature (see, for instance, Refs. [24,58,59]). This Appendix follows these derivations closely and provides additional background, which may be useful for readers less familiar with the field of hypothesis testing.

Let X∈[0,a] be a random variable with probability density p(x). We can formally write p(x)=−dP(X≥x)/dx, where $P\left(X\geq x\right)\equiv \int_{x}^{a}p\left(y\right)dy$ is the probability that *X* is larger or equal than *x*. We obtain from integration by parts

(A10) $$\begin{array}{ccc} \langle X^{2}\rangle =\int_{0}^{a}x^{2}p\left(x\right)dx & = & -{\left[x^{2}P\left(X\geq x\right)\right]}_{0}^{a}+2\int_{0}^{a}P\left(X\geq x\right)xdx\\ & = & 2\int_{0}^{a}P\left(X\geq x\right)xdx\\ & = & \frac{1}{2}\int_{0}^{2a}P\left(X\geq \frac{h}{2}\right)hdh, \end{array}$$

where we assume that *a* is finite [if a→∞ the above relation holds when $\lim_{a\to \infty }a^{2}P\left(X\geq a\right)=0$]. Finally, we can formally extend the above integral up to *∞* since P(X≥a)=0:

(A11) $$\langle X^{2}\rangle =\frac{1}{2}\int_{0}^{\infty }P\left(X\geq \frac{h}{2}\right)hdh.$$

Following Ref. [59], we now take $\epsilon =\theta_{\mathrm{est}}\left(\mu \right)-\theta_{0}$ and X=|ϵ|. We thus have

(A12) $$\mathrm{MSE}{\left(\theta_{\mathrm{est}}\right)}_{\mu ,\theta_{0}}={\langle |\epsilon |}^{2}\rangle =\frac{1}{2}\int_{0}^{\infty }P\left(\left|\epsilon \right|\geq \frac{h}{2}\right)hdh.$$

We express the probability as

$$\begin{array}{ccc} P\left(\left|\epsilon \right|\geq \frac{h}{2}\right) & = & P\left(\epsilon >\frac{h}{2}\right)+P\left(\epsilon \leq -\frac{h}{2}\right)\\ & = & P\left(\theta_{\mathrm{est}}\left(\mu \right)-\theta_{0}>\frac{h}{2}\right)+P\left(\theta_{\mathrm{est}}\left(\mu \right)-\theta_{0}\leq -\frac{h}{2}\right)\\ & = & \int P\left(\theta_{\mathrm{est}}\left(\mu \right)-\theta_{0}>\frac{h}{2}|\theta_{0}\right)p\left(\theta_{0}\right)d\theta_{0}+\int P\left(\theta_{\mathrm{est}}\left(\mu \right)-\theta_{0}\leq -\frac{h}{2}|\theta_{0}\right)p\left(\theta_{0}\right)d\theta_{0}. \end{array}$$

Next, we replace $\theta_{0}$ with $\theta_{0}+h$ in the second integral:

$$\begin{array}{ccc} P\left(\left|\epsilon \right|\geq \frac{h}{2}\right) & = & \int P\left(\theta_{\mathrm{est}}\left(x\right)-\theta_{0}>\frac{h}{2}|\theta_{0}\right)p\left(\theta_{0}\right)d\theta_{0}+\int P\left(\theta_{\mathrm{est}}\left(x\right)-\theta_{0}\leq \frac{h}{2}|\theta_{0}+h\right)p\left(\theta_{0}+h\right)d\theta_{0}\\ & = & \int \left(p\left(\varphi \right)+p\left(\varphi +h\right)\right)\left[\frac{p(\varphi )}{p(\varphi )+p(\varphi +h)}P\left(\theta_{\mathrm{est}}\left(x\right)-\varphi >\frac{h}{2}|\theta_{0}=\varphi \right)\right.+\\ & & \;+\left.\frac{p(\varphi +h)}{p(\varphi )+p(\varphi +h)}P\left(\theta_{\mathrm{est}}\left(x\right)-\varphi \leq \frac{h}{2}|\theta_{0}=\varphi +h\right)\right]d\varphi . \end{array}$$

We now take a closer look at the expression within the angular brackets and interpret it in the framework of hypothesis testing. Suppose that we try to discriminate between the two cases $\theta_{0}=\varphi$ (hypothesis 1, denoted $H_{1}$) and $\theta_{0}=\varphi +h$ (denoted $H_{2}$). We decide between the two hypothesis $H_{1}$ and $H_{2}$ on the basis of the measurement result *x* using the estimator $\theta_{\mathrm{est}}\left(x\right)$. One possible strategy consists in choosing the hypothesis whose value is closest to the obtained estimator. Hence, if $\theta_{\mathrm{est}}\left(x\right)\leq \varphi +h/2$, we assume $H_{1}$ to be correct and, otherwise, if $\theta_{\mathrm{est}}\left(x\right)>\varphi +h/2$, we pick $H_{2}$.

Let us now determine the probability to make an erroneous decision using this strategy. There are two scenarios that will lead to a mistake. First, our strategy fails whenever $\theta_{\mathrm{est}}\left(x\right)\leq \varphi +h/2$ when $\theta_{0}=\varphi +h$. In this case, $H_{2}$ is true, but our strategy leads us to choose $H_{1}$. The probability for this to happen, given that $\theta_{0}=\varphi +h$, is $P(\theta_{\mathrm{est}}\left(x\right)-\varphi \leq \frac{h}{2}|\theta_{0}=\varphi +h)$. To obtain the probability error of our strategy, we need to multiply this with the probability with which $\theta_{0}$ assumes the value φ+h, which is given by $p\left(H_{2}\right)=\frac{p(\varphi +h)}{p(\varphi )+p(\varphi +h)}$. Second, our strategy also fails if $\theta_{\mathrm{est}}\left(x\right)>\varphi +h/2$ for $\theta_{0}=\varphi$. This occurs with the conditional probability $P(\theta_{\mathrm{est}}\left(x\right)-\varphi >\frac{h}{2}|\theta_{0}=\varphi )$, and $\theta_{0}=\varphi$ with probability $p\left(H_{1}\right)=\frac{p(\varphi )}{p(\varphi )+p(\varphi +h)}$. The total probability to make a mistake is consequently given by

(A13) $$\begin{array}{ccc} P_{\mathrm{err}}\left(\varphi ,\varphi +h\right) & = & P\left(\theta_{\mathrm{est}}\left(x\right)-\varphi >\frac{h}{2}|H_{1}\right)p\left(H_{1}\right)+P\left(\theta_{\mathrm{est}}\left(x\right)-\varphi \leq \frac{h}{2}|H_{2}\right)p\left(H_{2}\right)\\ & = & \frac{p(\varphi )}{p(\varphi )+p(\varphi +h)}P\left(\theta_{\mathrm{est}}\left(x\right)-\varphi >\frac{h}{2}|\theta_{0}=\varphi \right)+\\ & & \;+\frac{p(\varphi +h)}{p(\varphi )+p(\varphi +h)}P\left(\theta_{\mathrm{est}}\left(x\right)-\varphi \leq \frac{h}{2}|\theta_{0}=\varphi +h\right), \end{array}$$

and we can rewrite Equation (A13) as

(A14) $$P\left(\left|\epsilon \right|\geq \frac{h}{2}\right)=\int_{-\infty }^{\infty }\left(p\left(\varphi \right)+p\left(\varphi +h\right)\right)P_{\mathrm{err}}\left(\varphi ,\varphi +h\right)d\varphi .$$

The strategy described above depends on the estimator $\theta_{\mathrm{est}}$ and may not be optimal. In general, a binary hypothesis testing strategy can be characterized in terms of the separation of the possible values of *x* into the two disjoint subsets $X_{1}$ and $X_{2}$ which are used to choose hypothesis $H_{1}$ or $H_{2}$, respectively. That is, if $x\in X_{1}$ we pick $H_{1}$ and otherwise $H_{2}$. Since one of the two hypotheses must be true, we have

(A15) $$\begin{array}{ccc} 1 & = & p\left(H_{1}\right)+p\left(H_{2}\right)\\ & = & \int_{X_{1}}dxp\left(x|H_{1}\right)p\left(H_{1}\right)+\int_{X_{2}}dxp\left(x|H_{1}\right)p\left(H_{1}\right)+\int_{X_{1}}dxp\left(x|H_{2}\right)p\left(H_{2}\right)+\int_{X_{2}}dxp\left(x|H_{2}\right)p\left(H_{2}\right)\\ & = & \int_{X_{1}}dxp\left(x|H_{1}\right)p\left(H_{1}\right)+\int_{X_{2}}dxp\left(x|H_{2}\right)p\left(H_{2}\right)+P_{\mathrm{err}}^{X_{1}}\left(H_{1},H_{2}\right), \end{array}$$

where the error made by such a strategy is given by

(A16) $$\begin{array}{ccc} P_{\mathrm{err}}^{X_{1}}\left(H_{1},H_{2}\right) & = & P\left(x\in X_{2}|H_{1}\right)p\left(H_{1}\right)+P\left(x\in X_{1}|H_{2}\right)p\left(H_{2}\right)\\ & = & \int_{X_{2}}p\left(x|H_{1}\right)p\left(H_{1}\right)dx+\int_{X_{1}}p\left(x|H_{2}\right)p\left(H_{2}\right)dx\\ & = & p\left(H_{1}\right)+\int_{X_{1}}\left[p\left(x|H_{2}\right)p\left(H_{2}\right)-p\left(x|H_{1}\right)p\left(H_{1}\right)\right]dx. \end{array}$$

This probability is minimized if $p\left(x|H_{2}\right)p\left(H_{2}\right)<p\left(x|H_{1}\right)p\left(H_{1}\right)$ for $x\in X_{1}$ and, consequently, $p\left(x|H_{2}\right)p\left(H_{2}\right)\geq p\left(x|H_{1}\right)p\left(H_{1}\right)$ for $x\in X_{2}$. This actually identifies an optimal strategy for hypothesis testing, known as the likelihood ratio test: if the likelihood ratio $p\left(x|H_{1}\right)/p\left(x|H_{2}\right)$ is larger than the threshold value $p\left(H_{2}\right)/p\left(H_{1}\right),$ we pick $H_{1}$, whereas, if it is smaller, we pick $H_{2}$. With this choice, the error probability is minimal and reads

(A17) $$\begin{array}{ccc} P_{\min}\left(H_{1},H_{2}\right) & = & \int_{X_{2}}\left[p\left(x|H_{1}\right)p\left(H_{1}\right)-p\left(x|H_{2}\right)p\left(H_{2}\right)\right]dx+\int_{X_{1}}\left[p\left(x|H_{2}\right)p\left(H_{2}\right)-p\left(x|H_{1}\right)p\left(H_{1}\right)\right]dx+\\ & & \;+\int_{X_{1}}p\left(x|H_{1}\right)p\left(H_{1}\right)dx+\int_{X_{2}}p\left(x|H_{2}\right)p\left(H_{2}\right)dx\\ & = & \frac{1}{2}-\frac{1}{2}\int \left|p\left(x|H_{1}\right)p\left(H_{1}\right)-p\left(x|H_{2}\right)p\left(H_{2}\right)\right|dx, \end{array}$$

where we used Equation (A15).

Applied to our case, we obtain

(A18) $$P_{\min}\left(\varphi ,\varphi +h\right)=\frac{1}{2}\left(1-\sum_{\mu }\left|\frac{p\left(\mu |\theta_{0}=\varphi \right)p\left(\varphi \right)}{p(\varphi )+p(\varphi +h)}-\frac{p\left(\mu |\theta_{0}=\varphi +h\right)p\left(\varphi +h\right)}{p(\varphi )+p(\varphi +h)}\right|\right).$$

This result represents a lower bound on $P_{\mathrm{err}}^{X_{1}}\left(\varphi ,\varphi +h\right)$ for arbitrary choices of $X_{1}$. This includes the case discussed in Equation (A13). Thus, using

(A19) $$P_{\mathrm{err}}\left(\varphi ,\varphi +h\right)\geq P_{\min}\left(\varphi ,\varphi +h\right)$$

in Equation (A14) and inserting back into Equation (A12), we finally obtain the Ziv–Zakai bound for the mean square error:

(A20) $$\mathrm{MSE}{\left(\theta_{\mathrm{est}}\right)}_{\mu ,\theta_{0}}\geq \frac{1}{2}\int_{0}^{\infty }hdh\int d\theta_{0}\left(p\left(\theta_{0}\right)+p\left(\theta_{0}+h\right)\right)P_{\min}\left(\theta_{0},\theta_{0}+h\right).$$

This bound can be further sharpened by introducing a valley-filling function [61], which is not considered here.
